# Beyond Hemostasis: Platelets’ Multifaceted Functions in Immune Responses

**DOI:** 10.3390/life16071209

**Published:** 2026-07-21

**Authors:** Woo Kyung Lee Doolittle, Elizabeth L. Walters, Robert W. Maitta

**Affiliations:** 1 Department of Hematology and Oncology, University Hospitals Seidman Cancer Center, Cleveland, OH 44106, USA; wookyung.doolittle@uhhospitals.org; 2 Case Western Reserve University School of Medicine, Cleveland, OH 44106, USA; elizabeth.walters@uhhospitals.org; 3 Department of Pathology, University Hospitals Cleveland Medical Center, Cleveland, OH 44106, USA

**Keywords:** platelets, innate immunity, adaptive immunity, receptors, granules, immune modulation, thromboinflammation

## Abstract

Platelets are anucleated cell fragments representing the second most abundant blood element in circulation, with approximately 100 billion new platelets released from the bone marrow daily in a healthy individual. For over a century, their hemostatic role was considered their primary function; however, the past few decades of research have revealed significant immunological functions in both innate and adaptive immunity. Through the release of mediators stored in platelet granules and the expression and realignment of a diverse array of surface receptors, platelets influence immune cells while simultaneously recognizing, reacting to, and phagocytosing offending pathogens such as viruses and bacteria. Their activation initiates signaling cascades that either amplify platelet responses or drive the activation, recruitment, migration, and maturation of immune cells to sites of infection. This narrative review synthesizes platelet biology, receptor signaling, granule physiology, and platelet interactions with innate and adaptive immune cells to provide an integrated perspective on platelet immunologic function. We examine platelets as frontline immune sentinels and evaluate their capacity to regulate both innate and adaptive immune responses, detailing specific receptors and granule contents as the mechanistic foundation for their immunologic roles. We further explore the molecular mechanisms by which platelets interact with pathogens and immune cells to drive pathogen clearance, inflammation modulation, and the interplay between thrombosis and immunity, including their contributions to chronic inflammatory disease and tumorigenesis.

## 1. Introduction

The description of platelets one hundred and fifty years ago by Dr. Giulio Bizzozero as unique peripheral blood elements represents one of the most important discoveries in medicine [1]. Their origin as vascular elements derived from megakaryocytes in the bone marrow was not established until the early 1900s [2], and their involvement in thrombosis and hemostasis would not become apparent until the 1960s [3], a reminder that nearly a century was needed to elucidate the basic platelet biology we now take for granted. Circulating mature platelets are small (2–5 µm) anucleated cellular elements that survive in circulation for up to 10 days and are maintained at a minimum count of approximately 150 × 10^9^/liter of peripheral blood [4,5]. Notably, megakaryocytes residing in extramedullary sites, particularly the lungs, contribute substantially to circulating platelet production, accounting for up to half of total output in murine models, with evidence of analogous hematopoietic progenitor activity in the adult human lung as well [6,7,8,9].

For decades, platelet biology was framed almost exclusively around hemostasis. Upon tissue injury, platelets activate and adhere to exposed subendothelial matrix via receptors binding collagen, von Willebrand factor (vWF), laminin, fibronectin, and thrombospondin, triggering signaling cascades that stabilize adherence and drive aggregation [10,11,12,13]. Integrin αIIbβ3-mediated platelet–platelet interactions then consolidate a fibrin-rich hemostatic plug further reinforced by FcγRIIa-coupled pathways and coordinated inside-out and outside-in integrin signaling that promotes granule release and morphological remodeling. Disruptions to this system, whether inherited, as in Bernard–Soulier syndrome (defects in GPIb-IX-V receptor complex) or Glanzmann thrombasthenia (intact coagulation cascade but impaired αIIbβ3 integrin function) [14], or acquired through medications or organ disease, compromise hemostasis and broader platelet physiology.

However, it is now well established that hemostasis is neither the only nor perhaps the primary function of platelets. Platelets mediate leukocyte and progenitor cell recruitment to injury sites, release both pro- and anti-inflammatory mediators that are characteristic of immune-competent cells, produce angiogenic factors and microparticles, phagocytose microorganisms, and directly activate immune effector cells [15,16,17,18,19,20]. The capacity to directly activate immune effector cells perhaps represents the strongest evidence that platelets are bona fide immune and inflammatory cells. This is further supported by sepsis models in which platelet activation, consumption, and immune cell interactions are consistently observed [21,22,23,24]. Platelets recognize pathogens through toll-like receptors (TLRs), driving auto-activation and enhancing neutrophil and monocyte functions at infection sites [25,26]. They also regulate adaptive immunity through T-cell modulation and endothelial cell activation, thereby bridging innate and adaptive immune responses [17]. These expanding non-hemostatic roles have motivated clinical translation efforts and early-phase trials targeting platelet–immune pathways [27,28,29].

This narrative review was conducted by systematically searching PubMed using terms encompassing platelet biology, immune receptors, granule physiology, innate immunity, adaptive immunity, thromboinflammation, and related pathologic conditions. Studies were selected and synthesized thematically based on their relevance to platelet immunologic function, from the authors’ integrative perspective. The review is organized to first establish the biological foundation of platelet structure and receptor biology, before examining in depth the mechanisms by which platelets modulate innate and adaptive immune responses, and their contributions to inflammatory disease and tumorigenesis.

## 2. Platelet Biology: From Biogenesis to Structure

Platelets originate in the bone marrow where megakaryocytes complete hematopoietic development through endomitosis [30], becoming large, polypoid cells with a single multilobulated nucleus positioned in extravascular spaces in close proximity to sinusoidal endothelial surfaces [30,31]. From there, megakaryocytes project cytoplasmic extensions rich in organelles into the sinusoidal lumen through a highly elaborated demarcation membrane system (DMS), which ultimately becomes the plasma membrane of nascent platelets [32,33]. The DMS fuses with trans-Golgi network (TGN)-derived vesicles interconnected with the endoplasmic reticulum, both enriched with phosphoinositides—signaling lipids essential to membrane trafficking, cytoskeletal organization, and cell signaling—that partition the megakaryocyte cytoplasm into individual platelet compartments [33,34]. Simultaneously, DMS-derived proplatelet (PPT) processes, guided by microtubules, transport granules and organelles into these compartments [35]. Each megakaryocyte can form up to eight PPTs, each up to 120 µm in length, collectively yielding approximately 1000–3000 platelets [36]. Proplatelet-like barbell structures then undergo fission to generate additional progeny, passing through a circulating “pre-platelet” intermediate (3–10 µm) that transitions into barbell forms before completing fission into mature platelets smaller than 3 µm [37,38]. Throughout this process, megakaryocytes continuously monitor the circulation and receive feedback signals regulating the pace of platelet production [39].

**Figure 1:**
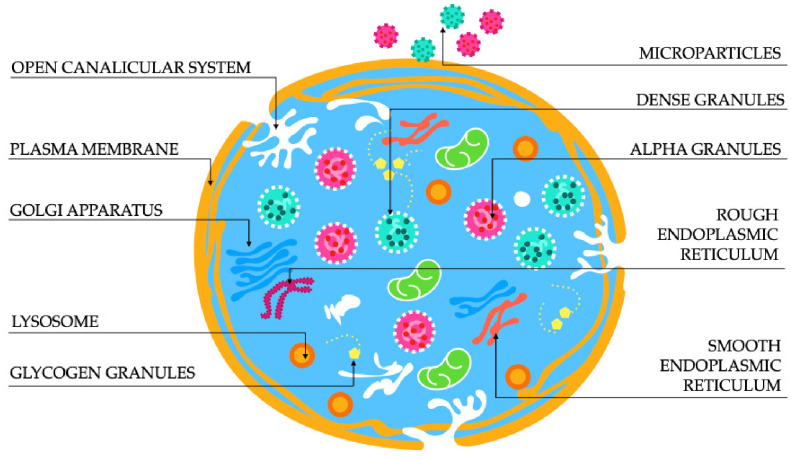
Structural organization of a resting platelet and its granule contents. At rest, platelets adopt a discoid morphology maintained by a marginal band of microtubules. Despite being anucleated, platelets contain a full complement of cellular organelles within a densely packed cytoplasm, including mitochondria, glycogen stores, peroxisomes, and an open canalicular system (OCS) that serves as a conduit for rapid granule content release upon activation. Three distinct granule subtypes are present: α-granules, the most abundant, storing adhesion proteins (fibrinogen, vWF, thrombospondin/TSP, GPIIb/IIIa), chemokines (PF4/CXCL-4, β-thromboglobulin/β-TG, SDF-1α), growth factors (VEGF, IGF, PDGF), and cytokines (TGF-β, CD40L); dense (δ-) granules, storing small bioactive molecules including serotonin (5-hydroxytryptamine/5-HT), ADP, ATP, Ca^2+^, polyphosphate, and histamine; and lysosomes (λ-granules), containing acid hydrolases (cathepsins, β-glucuronidase, hexosaminidase) and other proteases involved in receptor cleavage, matrix remodeling, and innate immune signaling. Together, these granule compartments constitute the primary secretory arsenal through which platelets exert hemostatic and immunomodulatory functions.

Mature platelets are discoid-shaped, anucleated elements approximately 2–5 µm in diameter with a lifespan of 7–10 days, that despite lacking a nucleus they contain virtually all structural attributes of immune-competent cells (Figure 1) [40,41,42]. Transmission electron microscopy reveals a densely packed cytoplasm containing mitochondria, microtubules, glycogen, peroxisomes, and three distinct granule subtypes: α-granules (most numerous), dense (δ-) granules, and lysosomes [43,44]. The platelet surface comprises an outer plasma membrane, open canalicular systems (OCS) serving as conduits for rapid granule release into the extracellular space, and a marginal band of microtubules underlying the plasma membrane [45]. Upon activation, platelets rapidly transition from their resting discoid shape to a spherical form with pseudopodia and filopodia projections driven by actin cytoskeletal remodeling, while simultaneously expressing or realigning surface receptors and releasing granular contents [42,46,47]. The OCS plays a critical role in this process, dramatically increasing the effective surface area available for receptor engagement and secretion [45]. Platelets vary in size according to their maturation state. Larger “reticulated” or “immature” platelets newly released from the bone marrow, have high RNA content and are metabolically more active. They circulate for only 24–36 h before progressive RNA degradation, and volume reduction yield the smaller ‘mature’ platelet form [48].

In summary, the megakaryocyte-to-platelet developmental sequence is a tightly regulated process that endows platelets with a rich internal architecture—a dense granule payload and a dynamically remodeled surface receptor repertoire—precisely suited for their dual roles in hemostasis and, as the following sections detail, immune modulation.

## 3. Immune-Sensing Platelet Receptors

Platelets occupy a dual and at times contradictory position as both host defense effectors and potential initiators of immunopathology [49]. This functional duality is largely mediated through two major systems: the complement system and a broad repertoire of pattern recognition receptors (PRRs) [50]. Together these systems allow platelets to sense an extraordinarily wide range of threats from bacterial cell wall components and viral nucleic acids to host-derived damage signals, and to respond without delay given their lack of a nucleus [51,52].

**Figure 2:**
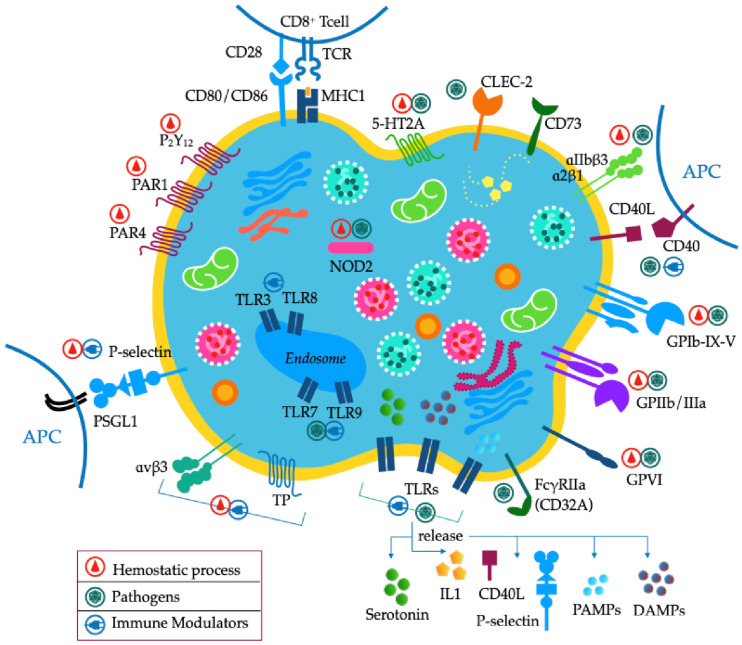
Diagram of select immune-responsive platelet surface and intracellular receptors categorized by their primary functional roles. Hemostasis-only receptors (surface): protease-activated receptors PAR1 and PAR4, which respond to thrombin cleavage, and the purinergic ADP receptor P2Y_12_, which amplifies platelet aggregation. Pathogen recognition receptors (surface): FcγRIIa (CD32a), which detects IgG immune complexes; and C-type lectin-like receptor 2 (CLEC-2), which recognizes pathogen-associated carbohydrates and lipids including dengue virus and HIV. Dual hemostatic and pathogen-sensing receptors (surface): the GP1b-IX-V complex and GPVI, which mediate both subendothelial matrix adhesion and immune cell interactions; integrin receptors αIIbβ3 and α2β1, which support platelet–leukocyte aggregates and NETosis; and the serotonin 2A receptor (5-HT2A), which links dense granule serotonin release to immune cell modulation. Immune modulatory receptors (surface): Toll-like receptors (TLRs 1, 2, 4, 5, 6, and 10), which sense bacterial and fungal PAMPs; CD40L (CD154), which co-stimulates B cells, dendritic cells, and T cells; and P-selectin (CD62P), which mediates platelet–leukocyte tethering and leukocyte recruitment via PSGL-1 binding. Intracellular receptors (endosomal/lysosomal compartments): TLR3, TLR7, TLR8, and TLR9, which sense viral and bacterial nucleic acids in the acidic endo-lysosomal environment; and NOD2, which recognizes bacterial peptidoglycans to promote platelet aggregation and innate immune signaling.

Platelets express an array of immune-sensing and adhesion receptors on their surface and within intracellular compartments (Figure 2). These include leucine-rich repeat receptors (GP1b-IX-V complex) [53], TLR1–10 [54,55], immunoglobulin superfamily receptors bearing either immune-receptor tyrosine-based activation motif (ITAM) or inhibitory motif (ITIM) signaling domains (GPVI, FcγRIIa/CD32a, FcεRI, PECAM-1/CD31, TLT-1), integrins (αIIbβ3, α2β1, αvβ3) [56], selectins (P-selectin/CD62P), tetraspanins (CD9, CD63, CD151, Tspan9) [57], G protein-coupled receptors (P2Y_1_, P2Y_12_, PAR1, PAR4), and co-stimulatory molecules (CD40, CD40L/CD154) [58,59,60]. Engagement of these receptors initiates signaling cascades that drive granule release, receptor realignment, extracellular vesicle production, and intercellular communication with virtually every arm of the immune system [41].

Beyond direct receptor-mediated signaling, platelets release extracellular vesicles, including ectosomes (microvesicles) shed from the plasma membrane and exosomes derived from the endosomal system, that transport nucleic acids, proteins, and lipid mediators to recipient immune cells, extending platelet influence beyond sites of direct contact communication [52,61]. Critically, over 300 bioactive proteins have been identified in platelet granules alone, spanning functions in immunity, inflammation, angiogenesis, and cell growth [6,15], underscoring the breadth of the platelet–immune-sensing apparatus.

The downstream consequences of receptor engagement follow a coordinated two-pronged strategy. First, activated platelets generate a physical containment barrier that limits pathogen dissemination [54]. Simultaneously, they release targeted activators of neutrophils, monocytes, lymphocytes, and dendritic cells (DCs) calibrated to the nature of the pathogenic threat [62]. After phagocytosing infectious agents, platelets can also then either directly present antigens or activate professional antigen-presenting cells (APCs), bridging innate and adaptive immune responses [16,63]. NOD-like receptors (NOD1, NOD2, NLRPs) sensing bacterial peptidoglycans promote platelet aggregation and clot formation [64], while C-type lectin receptors such as CLEC-2, recognizing carbohydrates, proteins, and lipids on pathogens, further mediate aggregation responses [65,66,67]. Pathogen-associated molecular patterns (PAMPs) and damage-associated molecular patterns (DAMPs) from damaged host cells are recognized by TLRs to initiate both immune and inflammatory responses [68,69].

In summary, the platelet–immune-sensing receptor repertoire is remarkable in both breadth and functional consequence, encompassing surface PRRs, intracellular nucleic acid sensors, Fc receptors, adhesion molecules, and co-stimulatory ligands that collectively position platelets as versatile first-responders capable of detecting, containing, and coordinating responses to a wide variety of immunologic threats. The following subsections address each receptor class in detail.

### 3.1. Leucine-Rich Repeat Receptors (LRR) and Toll-Like Receptors (TLRs)

LRR-containing receptors are found on the cell surface, intracellularly, or as secreted proteins. The LRR domain—characterized by recurring three-dimensional arrangements of 20–30 amino acid sequences rich in leucine, with each motif expressing both a β-strand and α-helix linked by loops—enables protein–protein interactions through which surface LRR proteins recognize pathogens and mediate cell-to-cell communication among immune cells [70,71]. On platelets, the most recognizable and abundant LRR-containing receptor is the GP1b-IX-V complex, which is essential to hemostasis and thrombosis, and shares the LRR architecture found in TLR extracellular domains, structurally linking these two receptor families [59,72,73].

In the human innate immune system, TLR1–10 are PRRs expressed on DCs, B cells, macrophages, platelets, fibroblasts, and epithelial cells [55]. Platelets express TLRs both on their surface (TLR1, TLR2, TLR4, TLR5, TLR6, and TLR10) and within endosomal compartments (TLR3, TLR7, TLR8, and TLR9), enabling detection of a broad range of PAMPs and DAMPs [54,55]. All TLRs share a LRR ligand-binding ectodomain, a single transmembrane domain, and a cytoplasmic Toll/IL-1 receptor (TIR) domain that mediates downstream signaling. Upon ligand binding, TLRs form an M-shaped homodimer, with the exception of TLR2, which signals only as a heterodimer with either TLR1 or TLR6 [74,75], a pairing that determines the specific microbial PAMPs recognized [76] and is particularly important for the phagocytic capabilities of platelets [77].

At the platelet surface, TLR4 activation by LPS, an outer membrane component of Gram-negative bacteria, triggers neutrophil activation and neutrophil extracellular trap release (NETosis) through a mechanism distinct from classical platelet aggregation [78], while TLR2 stimulation promotes platelet aggregation via phosphoinositide 3-kinase (PI3K) signaling, facilitating bacterial detection [77]. Within endosomal compartments, TLRs specialize in nucleic acid sensing: TLR3 detects double-stranded RNA [79,80], TLR7 recognizes single-stranded RNA [81,82], and TLR9 binds unmethylated CpG DNA motifs, with platelet-specific T granules serving as organizational platforms for TLR9 signaling [83,84]. For example, the TLR7-dependent platelet response to influenza virus, in which platelet activation induces C3 release that subsequently drives neutrophil-mediated NETosis, is a pathway implicated as a central driver of acute lung injury and thrombotic sequelae frequently observed in severe influenza [85,86].

A particularly significant downstream consequence of platelet TLR activation is assembly of the NLRP3 inflammasome, a multiprotein complex that enables platelets to synthesize and process IL-1β and IL-18 de novo despite being anucleate [87]. TLR4 activation by LPS provides the priming signal, inducing IL-1β pre-mRNA splicing and protein synthesis via MyD88–IRAK1–TRAF6 signaling, while a second signal—such as ATP acting on P2X7 receptors or dengue virus engagement via DC-SIGN—triggers inflammasome assembly, caspase-1 activation, and IL-1β processing [87,88,89,90]. Critically, platelets not only produce IL-1β themselves but amplify inflammasome activation in neighboring innate immune cells, enhancing NLRP3 capacity in macrophages and neutrophils and licensing IL-1β production in monocytes through a contact-independent mechanism [91]. The platelet NLRP3 inflammasome has been implicated in sickle cell disease [92], acute coronary syndrome [93], sepsis [94] and viral hemorrhagic fever [87], establishing it as a key amplifier through which platelet TLR signaling propagates inflammatory responses.

Consequently, the LRR–TLR axis equips platelets with a sophisticated, dual-compartment pathogen-sensing system capable of detecting extracellular bacterial components and intracellular viral nucleic acids alike, and of translating these signals into coordinated innate immune responses ranging from NETosis and complement activation to inflammasome-driven cytokine amplification.

### 3.2. Cell Adhesion Molecules (CAMs)

CAMs are critical mediators of immune function, operating through homophilic or heterophilic receptor configurations that determine their roles in immune recognition, cell adhesion, and intercellular signaling [95]. They mediate cell-to-cell interactions and binding to the extracellular matrix, and are subdivided into five families: selectins, integrins, immunoglobulin superfamily (IgSF) proteins, cadherins, and mucins. On platelets, CAMs encompass a functionally diverse set of proteins that collectively mediate leukocyte recruitment, pathogen containment, and bidirectional communication between innate and adaptive immunity.

#### 3.2.1. Immunoglobulin Superfamily (IgSF) Receptors

IgSF receptors are a group of membrane and soluble proteins characterized by multiple immunoglobulin-like domains that mediate cell-to-cell contact necessary for development, immune recognition, and cell-fate determination [96,97]. Platelet IgSF receptors include GPVI, FcγRIIa, FcεRI, PECAM-1 (CD31), and TLT-1, each carrying either ITAM or ITIM signaling motifs that, respectively, drive or dampen platelet activation [98,99]. Junctional adhesion molecules (JAMs), present at intercellular junctions of endothelial and epithelial cells as well as on leukocyte and platelet surfaces, also belong to this family; JAM-A on platelets are particularly important to early vascular injury repair and atherosclerotic vascular changes [100].

##### GPVI

GPVI is a 62 kDa transmembrane receptor expressed exclusively on platelets and megakaryocytes that serves as the principal signaling receptor for extracellular matrix components including collagen, laminin, and nidogen [101]. It forms a complex with the Fc receptor γ-chain (FcRγ) via a disulfide bond, whose ITAMs undergo phosphorylation upon receptor engagement to initiate platelet activation. Exposure to collagen, fibrinogen, and fibrin polymers triggers GPVI-mediated ITAM phosphorylation by Src family kinases (Lyn and Fyn), enabling Syk recruitment and activation, followed by assembly of the LAT–SLP-76 signalosome that orchestrates PLCγ2, PI3K, and Btk activation, culminating in calcium flux, integrin activation, granule secretion, and procoagulant surface formation [102,103,104]. Beyond hemostasis, GPVI activation by fibrin, fibrinogen, and laminin during immune responses supports platelet adhesion and signaling independently of classical thrombus formation, enabling vessel repair in inflamed tissues [105]. In pneumonia-associated sepsis, GPVI enhances leukocyte-mediated bacterial clearance [106], while conversely, in acute lung injury, GPVI signaling promotes neutrophil recruitment, platelet–neutrophil aggregation, and NET release, driving thrombo-inflammatory injury [107], illustrating the context-dependent duality of GPVI immune function.

##### FcγRIIa

FcγRIIa (CD32a) is a platelet ITAM-bearing low-affinity IgG receptor expressed on human but not murine cells, composed of two extracellular immunoglobulin-like domains, a single transmembrane segment, and a cytoplasmic ITAM tail containing two YXXL motifs [108]. Although it binds monomeric IgG with low affinity, FcγRIIa efficiently recognizes Fc region of antibodies within immune complexes, initiating a robust ITAM-dependent signaling cascade comparable to GPVI, with Syk recruitment as a central event [98,109]. This pathway drives platelet activation but also engages negative feedback through ADAM family metalloproteinases and the Ca^2+^-dependent protease calpain, resulting in GPVI shedding and proteolytic cleavage of FcγRIIa’s cytoplasmic ITAM domain, attenuating further signaling [99]. Through its capacity to sense immune complexes, FcγRIIa positions platelets as active participants in humoral immune surveillance, discussed further in Section 5.

##### FcεRI

Platelets express the high-affinity IgE receptor FcεRI—primarily known on mast cells, basophils, and APCs—where it drives IgE-mediated platelet activation, aggregation, and release of inflammatory mediators [110,111]. Platelets additionally express the low-affinity FcεRII (CD23), which downregulates FcεRI activity and contributes to IgE storage [112]. ITAM signaling downstream of FcεRI mediates serotonin and RANTES release from activated platelets, amplifying allergic and inflammatory responses [113].

##### PECAM-1

PECAM-1 (CD31) is expressed on platelets, granulocytes, T cells, and monocytes, and mediates platelet–endothelium adhesion, endothelial junction integrity, and leukocyte transmigration to sites of infection and antigenic exposure [114,115]. Structurally, its cytoplasmic tail contains ITIMs whose phosphorylation creates docking sites for SHP-1 and SHP-2 phosphatases, which inhibit ITAM-driven platelet activation pathways—making PECAM-1 an important counterbalance to activated IgSF receptors [104,116,117,118].

##### TLT-1

TLT-1 is found exclusively in platelet α-granules and megakaryocytes, with an extracellular domain homologous to the triggering receptors expressed on myeloid cells (TREM) family [119,120]. Upon platelet activation, TLT-1 is surface-expressed where it serves dual immunomodulatory roles: soluble TLT-1 dampens leukocyte activation during sepsis through competitive inhibition of TREM-1 ligand binding [121], while membrane-bound TLT-1 facilitates controlled leukocyte transmigration and fibrinogen deposition at sites of inflammation in an acute lung injury mouse model [122] and drives platelet–monocyte aggregation to promote IL-10-producing B cells in tuberculosis [123].

#### 3.2.2. Integrins

Integrins are non-covalently bound transmembrane heterodimers composed of α- and β-subunits that link extracellular ligands to intracellular signaling pathways, allowing platelets to recruit leukocytes coupling innate and adaptive immune responses [56,124,125]. Among platelet integrins, αIIbβ3 (GPIIb/IIIa) is the most abundant, undergoing inside-out activation to bind fibrinogen, vWF, and matrix proteins [126]. Beyond mediating aggregation, αIIbβ3 supports platelet–neutrophil aggregate formation via fibrinogen bridging to Mac-1 [127], promotes NETosis [128], and facilitates platelet delivery of CD40L to DCs and B cells, enhancing antigen presentation, class-switch recombination, and CD8^+^ T cell responses [129]. Additional integrins—α2β1, α5β1, α6β1, and αvβ3—contribute to platelet adhesion to collagen, fibronectin, laminin, and vitronectin within inflamed tissues, expanding the integrin-mediated immune footprint beyond αIIbβ3.

#### 3.2.3. Selectins

Selectins are adhesion receptors distributed across platelets (P-selectin/CD62P), endothelial cells (E-selectin), and lymphocytes (L-selectin) [130]. P-selectin is stored within α-granules at rest and rapidly translocates to the platelet surface upon activation, where it mediates platelet–leukocyte interactions and leukocyte recruitment to injury sites through binding PSGL-1 on leukocytes [131]. E-selectin and L-selectin coordinate leukocyte rolling and trafficking, supporting broader vascular immune responses [132,133].

### 3.3. Tetraspanins

Tetraspanins are four-pass transmembrane proteins that organize membrane microdomains and regulate the trafficking, clustering, and signaling function of associated receptors [57]. The platelet tetraspanin family includes CD63, CD9, CD151, and Tspan9 [134]. CD63, stored in α-granules at rest, migrates to the platelet surface upon activation where it serves as an essential cofactor for P-selectin-mediated leukocyte rolling and recruitment in a peritonitis mouse model [135]. Notably, platelet-derived αIIbβ3/CD9-enriched membrane tethers exacerbate severe inflammation by redistributing the αIIbβ3/CD9 pool from platelets to the leukocyte–endothelium interface, driving neutrophil activation and infiltration—a mechanism that may explain the paradoxical coexistence of thrombo-inflammation and bleeding in critical illness [136].

### 3.4. G-Protein-Coupled Receptors (GPCRs)

GPCRs are seven-pass transmembrane proteins that transmit extracellular signals through heterotrimeric G proteins [137]. On platelets, the principal GPCRs are the purinergic ADP receptors P2Y_1_ and P2Y_12_, and the thrombin-responsive protease-activated receptors PAR1 and PAR4 [138]. Signal initiation involves ligand binding—or in the case of PARs, proteolytic cleavage by thrombin exposing a tethered ligand—activating Gα subunits (Gα_13_, Gαq, or Gαi) and dissociation of the Gβγ dimer [139]. Gα_13_ activates the RhoA–ERK1/2 pathway driving shape change [140], Gαq targets PLCβ to generate IP_3_ and DAG triggering calcium flux and granule secretion [141], while Gαi and Ca^2+^ reinforce platelet activation through the SFK–PI3K–AKT axis [142].

Beyond their hemostatic roles, platelet GPCRs actively shape immune responses. ADP promotes monocyte activation in a platelet-dependent manner, an effect blocked by P2Y_1_ or P2Y_12_ antagonists [143]; and in chronic hepatitis B, combined P2Y_12_ inhibition with aspirin reduces intrahepatic HBV-specific CD8^+^ T cell infiltrates and mitigates immune-mediated hepatocarcinogenesis [144]. Thrombin–PAR1 signaling enhances IFN-β expression during ssRNA viral infection, contributing to innate antiviral defense [145], while PAR4 promotes platelet–leukocyte interactions and granule release, and its inhibition augments Treg activation, underscoring its pro-inflammatory role [146,147].

In summary, platelet CAMs, tetraspanins, and GPCRs collectively constitute a sophisticated surface interface through which platelets physically engage, recruit, and modulate virtually every category of immune effector cell. The ITAM/ITIM balance within IgSF receptors, the bridging functions of integrins and selectins, the membrane-organizing roles of tetraspanins, and the immune-modulatory outputs of GPCR signaling together enable platelets to fine-tune inflammatory responses across a wide range of pathologic contexts.

## 4. Platelet Granules and Their Mediators

Platelets store the vast majority of their bioactive cargo as pre-packaged proteins within three types of intracellular secretory granules: α-granules, dense (δ-) granules, and lysosomal (λ-) granules (Table 1) [16]. Upon activation, granules translocate to the platelet surface and release their contents through the OCS into the extracellular space, generating cascades of immune and hemostatic signaling events [148]. Platelets additionally release bioactive molecules through direct membrane protein shedding—a process termed the physiologic “sheddome”—further expanding the range of secreted mediators [54,149]. Both α- and δ-granules initiate biogenesis in the trans-Golgi network (TGN) and endosomal compartments of megakaryocytes, progressing through maturing multivesicular bodies (MVBs) transported into proplatelet extensions before being packaged into nascent platelets [35,148]. At the TGN, clathrin- and AP1-associated vesicles fuse with early endosomes [150], while plasma membrane-derived vesicles form through AP2-mediated endocytosis converging on the same compartment [151]. α-granule maturation proceeds within MVBs and requires the BEACH-domain proteins VPS33B [152], VPS16B [153], and NBEAL2 [154], with mutations in any of these resulting in α-granule deficiency syndromes. δ-granules are lysosome-related organelles derived from the endosomal system via BLOC-1/2 complexes and an AP3-dependent trafficking pathway [155], and similarly undergo final maturation within MVB intermediates [156].

### 4.1. Alpha (α-) Granules

α-granules are the most abundant secretory granule in platelets, numbering approximately 50–80 per platelet [211,212], and store a remarkably diverse arsenal of mediators spanning adhesion, inflammation, immune defense, and tissue repair [63,213]. Their cargo includes adhesion proteins (fibrinogen, vWF, P-selectin, GPIIb/IIIa), chemokines (PF4/CXCL-4, NAP-2/CXCL-7, IL-8/CXCL-8, MIP-1α/CCL-3, RANTES/CCL-5), cytokines (TGF-β, soluble CD40L), growth factors (PDGF, VEGF), and microbicidal proteins (kinocidins, thrombocidins 1 and 2, α-defensin 1). Upon activation, vWF supports leukocyte tethering and rolling by promoting adhesion of leukocytes to platelets and endothelial cells [214], while P-selectin attracts PMNs and initiates rolling adhesion through PSGL-1 binding [215], and GPIIb/IIIa promotes platelet–leukocyte and platelet–endothelial interactions via fibrinogen and vWF bridging [216]. The full scope of chemokine and cytokine targets released from α-granules—spanning PMNs, monocytes, lymphocytes, and endothelial cells—is summarized in Table 1.

### 4.2. Dense (δ-) Granules

Dense granules are small electron-dense organelles, typically 3–6 per platelet [217,218], whose contents are subdivided into bioactive amines (serotonin, histamine), bioactive ions (Ca^2+^, PO_3_^−^), nucleotides (ADP, ATP, GTP), and inorganic polyphosphates [219]. Histamine acts as a pro-inflammatory modulator of endothelial cells, leukocytes, and lymphocytes [200,220,221], while serotonin modulates monocyte and T cell activation [201,202,203]. Ca^2+^ and phosphate ions support numerous systemic functions including cell adhesion and the coagulation cascade, and ADP, ATP, and GTP activate purinergic receptors on immune cells to amplify inflammatory responses [16,63]. Notably, ATP released from δ-granules also provides the second signal for NLRP3 inflammasome assembly via P2X7 receptor engagement, linking δ-granule secretion directly to the inflammasome pathway described in Section 3.1. Deficiencies in δ-granules result in “delta-storage pool diseases,” exemplified by Hermansky–Pudlak syndrome, with associated bleeding and immune dysregulation [222].

### 4.3. Lysosomal (λ-) Granules

Lysosomal granules are membrane-bound organelles, typically 1–3 per mature platelet [148], enriched with acid hydrolases—including cathepsins, arylsulfatase, hexosaminidase, β-glucuronidase, β-galactosidase, and acid phosphatase—and bearing surface markers CD63 and LAMP-1/2 shared with δ-granules [223]. Unlike α- and δ-granules, which are released in the first wave of platelet exocytosis, lysosomes require stronger agonist stimulation and release their contents in a delayed second wave [224], contributing to receptor cleavage, fibrin degradation, extracellular matrix remodeling, and vascular repair [225]. Their degradative and phagocytic properties, while less well characterized than those of other granule types, share enzymatic composition with lysosomes in nucleated immune cells and involve unconventional secretory pathways [224,226].

Platelet lysosomes are tightly linked to a constitutively active autophagy pathway upregulated under nutrient deprivation or mTOR inhibition via a PI3K-dependent mechanism [227,228]. Core autophagy proteins—ATG5, ATG7, LC3, Beclin 1, and PIK3C3—are expressed in platelets, and their disruption has marked functional consequences: heterozygous Beclin 1 loss impairs platelet aggregation and adhesion [228], while ATG7 deletion disrupts megakaryocyte and platelet granule packaging, causing severe bleeding phenotypes [227]. Autophagy induction has also been shown to be protective in immune thrombocytopenia [229].

Platelet lysosomes intersect with innate immunity through endosomal TLR signaling [230]. The acidic endo-lysosomal environment is required to activate TLR7 and TLR9 following internalization of pathogen-derived nucleic acids [50], and this endosomal TLR activation coordinates downstream systemic immune responses, including platelet–neutrophil interactions, NETosis, and complement cascade amplification through C3 surface expression [78,86].

To conclude, the three granule types of platelets constitute a tiered and highly coordinated secretory arsenal: α-granules deliver the broadest immunomodulatory payload targeting virtually every arm of the immune system; δ-granules provide rapid-release small molecules that amplify local inflammatory and inflammasome responses; and lysosomal granules contribute degradative enzymes and serve as the intracellular platform through which platelets sense and respond to internalized pathogens via endosomal TLR signaling. Together, these compartments translate platelet activation into a sustained, multifaceted immune response.

## 5. Platelets as Immune Modulators

Platelet activation initiates a series of complex intracellular signaling cascades that prime platelets to respond to the specific nature of the detected insult or threat [104]. These signals drive rapid morphological transformation—from a resting discoid form to a spherical shape with filopodia and lamellipodia projections—that expand the platelet surface area and enable dynamic sampling of the immediate microenvironment [63]. This morphological change, driven by actin cytoskeletal reorganization and polymerization, is not merely structural: it physically positions platelets to engage immune effector cells, release granule contents in a directional manner, and establish direct intercellular contacts that bridge innate and adaptive immunity. The following subsections examine the mechanisms through which activated platelets modulate innate and adaptive immune responses.

### 5.1. Innate Immune Modulation

#### 5.1.1. Platelet–Neutrophil Interactions

Platelets interact with neutrophils through multiple complementary mechanisms to coordinate recruitment, activation, and effector function at sites of injury and infection [231,232]. At the earliest stage, platelets adhere to neutrophils and guide their extravasation to sites of inflammation while simultaneously preventing premature neutrophil cell death and dynamically regulating their immunometabolic state [233,234]. Once in contact, platelets trigger neutrophil oxidative bursts and prime NET release, which further amplifies the local procoagulant environment by expanding the cell surface available for coagulation factor binding [235,236].

The molecular basis of platelet–neutrophil engagement begins with P-selectin binding to PSGL-1 on neutrophils, enabling tethering, rolling, and activation at sites of inflammation [237,238]. This initial contact is reinforced and extended by platelet-derived serotonin metabolite 5-hydroxyindoleacetic acid (5-HIAA), which acts as a ligand for GPR35 on neutrophils to enhance their recruitment [239]. Similarly, platelet TLR4 activation promotes platelet–neutrophil aggregate formation, augmenting neutrophil adhesion to the endothelium and transmigration [78,240], while activated platelet-derived nanovesicles independently recruit neutrophils as anti-tumor effectors upon detection of malignant transformation [241]. Not all platelet–neutrophil interactions, however, are amplificatory: in acute toxic liver injury, platelet-initiated CLEC-2 signaling paradoxically worsens outcome by reducing TNF-α production and limiting reparative neutrophil recruitment to the liver, illustrating the context-dependence of these interactions [242].

Past initial recruitment, platelets directly regulate neutrophil activation through formation of platelet–neutrophil complexes (PNCs), a process representing a positive bidirectional feedback loop in which platelets release neutrophil activators while activated neutrophils in turn release factors that further enhance platelet activation [243]. Within PNCs, neutrophil Mac-1 binds platelet GPIbα, JAM-3, and fibrinogen bridged through αIIbβ3, while neutrophil LFA-1 engages platelet ICAM-2. Additionally, neutrophil SLC44A2 binds activated platelet αIIbβ3 under shear conditions, promoting NETosis [41,127,128,244]. Platelet-derived chemokines PF4/CXCL4 and NAP-2/CXCL7, together with neutrophil-driven myeloperoxidase (MPO), mediate key activating signals within these complexes [105], while platelet-derived arachidonic acid generates leukotrienes LTB4 and LTA4 that feed back to amplify neutrophil activation [245]. MPO and platelet-derived serotonin additionally promote neutrophil degranulation, upregulate the adhesion molecule CD11b, and induce further MPO and hydrogen peroxide release, sustaining the inflammatory loop [246]. Counterbalancing these amplificatory mechanisms, platelet ACKR3/CXCR7 activation dampens platelet-neutrophil aggregate formation and thromboinflammatory secretion through generation of antithrombotic lipids and cAMP/PKA-mediated platelet inhibition, representing a protective checkpoint against excessive platelet–PMN-driven tissue damage [247,248,249].

#### 5.1.2. Platelet–Monocyte/Macrophage Interactions and Inflammatory Resolution

Beyond neutrophils, platelets modulate monocytes and macrophages that shape the quality and duration of innate immune responses. As described in Section 3.4, ADP released from activated platelets promotes monocyte activation in a P2Y_12_-dependent manner [143], while platelet-derived serotonin modulates monocyte cytokine and chemokine production through engagement of multiple 5-HT receptor subtypes [201,202,203]. Platelet-derived PDGF drives monocyte and macrophage differentiation [165,166], and TGF-β released from α-granules promotes macrophage phenotypic switching toward anti-inflammatory M2-like state [188]. Notably, platelets orchestrate resolution of pulmonary inflammation through Treg cell repositioning and macrophage education [250], underscoring their active role not merely in initiating but in resolving innate immune responses. TLT-1-mediated platelet–monocyte aggregate formation further extends this regulatory capacity by promoting IL-10-producing B cells in tuberculosis [123], establishing a mechanistic link between platelet–monocyte interactions and downstream humoral immunity.

Thus, platelet innate immune modulation is executed through an organized, receptor-driven program encompassing direct cellular contacts, granule-mediated paracrine signaling, extracellular vesicle communication, and NET regulation. The platelet–neutrophil axis dominates acute inflammatory amplification, while platelet–monocyte/macrophage interactions contribute to inflammatory resolution and transition toward adaptive immunity—themes that are explored in the following section.

### 5.2. Adaptive Immune Modulation

Adaptive immunity, mediated by antigen-specific memory T and B cells, follows the initial innate immune response to ensure a robust and long-lasting defense against pathogens. Platelets facilitate lymphocyte trafficking to lymphoid organs, enabling antigen presentation while simultaneously influencing lymphocyte maturation and polarization [51,251].

#### 5.2.1. Platelets in T Cell Function and Differentiation

Lymph nodes serve as hubs for antigen presentation and lymphocyte activation, with APCs such as lung-derived DCs migrating to the spleen for T cell priming during influenza infection [252]. Platelet CLEC-2 is critical for lymphatic vascular development [253] and maintenance of lymph node architecture—its deletion leads to lymph node fibrosis and blood backfilling, impairing immune cell recirculation and secondary immune responses [254,255]. Similarly, platelets actively guide immune cell recruitment to sites of antigen exposure [60]. Once activated, platelets translocate P-selectin to promote T cell rolling into high endothelial venules (HEVs), enabling lymphocyte entry into secondary lymphoid organs [256,257]. This is demonstrated in infectious models showing that activated platelets are required for intrahepatic accumulation of virus-specific cytotoxic T cells in acute viral hepatitis [258], that PF4–CXCR3 signaling is essential for T cell recruitment to the brain in malaria [168], and that platelet depletion reduces antigen-specific CD4^+^ T cells and improves outcomes in murine experimental autoimmune encephalomyelitis [259].

Platelets influence antigen presentation both directly and indirectly. They promote DC differentiation from monocytes via P-selectin/PSGL-1 signaling [260] and enhance DC maturation and type I interferon production through CD40L [261]. Platelets themselves directly present antigen via MHC class I, with upregulation of platelet MHC class I enabling internalization, processing, and presentation of antigens to CD8^+^ T cells—a mechanism that suppresses T cell proliferation and limits functional responses during sepsis [262,263]. Additionally, platelets shuttle complement 3-opsonized *Listeria monocytogenes* to splenic CD8α^+^ DCs via GPIb and complement-dependent mechanisms to trigger adaptive immune responses [264], with analogous trafficking observed with adenovirus [265]. Platelets also express co-stimulatory molecules CD80 and CD86, which bind CD28 on T cells to enhance activation, proliferation, and cytokine production [262].

Platelets are active orchestrators of T cell-dependent immunity through CD40L. Platelet-derived CD40L restores CD8^+^ T cell activity in CD40L-deficient mice and its depletion reduces cytotoxic T cell responses [266,267]. Remarkably, platelet CD40L alone can drive T cell priming and induce B cell isotype switching during viral infection even under low antigen exposure [129,268]. In the liver, platelets serve as intravascular docking sites via CD44 on sinusoidal hyaluronan, enabling CD8^+^ T cells to crawl along sinusoids, extend protrusions, and survey hepatocytes for antigens without exiting the bloodstream—a mechanism through which antiplatelet therapy mitigates inflammation and prevents hepatocellular carcinoma development [144,269].

Platelets also shape T cell polarization by modulating the cytokine milieu. Naïve CD4^+^ T helper cells differentiate into Th1, Th2, Th17, and Tregs based on the prevailing cytokine environment [270], and platelets—as major sources of plasma cytokines and chemokines—actively sculpt this environment. Platelet exposure to *Candida albicans* via GPIb triggers Dickkopf-1 (Dkk1) release, promoting Th2 and Th17 polarization and coordinating a protective allergic immune response against fungal airway infection [271]. As the primary circulating reservoir of TGF-β, platelets drive Treg polarization through its release [272], consistent with the reduced Treg counts observed in thrombocytopenia [273]. Platelet-derived microparticles further stabilize Tregs by inhibiting IL-17 and IFN-γ production through P-selectin–PSGL-1 signaling [274], while direct physical platelet–Treg interactions modulate the Treg transcriptome to promote IL-10 and TGF-β secretion [250]. Collectively, platelets regulate T cell biology at every level—guiding migration, enabling activation, and fine-tuning functional fate.

#### 5.2.2. Platelet–B Cell and Humoral Immune Interactions

Platelets actively shape humoral immunity through two principal mechanisms: FcγRIIa-mediated immune complex sensing and CD40L/PF4-dependent B cell co-stimulation [275].

As the largest pool of FcγRIIa in humans, platelets bind circulating IgG-containing immune complexes, facilitating their clearance [251]. Cross-linking of FcγRIIa by IgG-opsonized pathogens or pathological autoantibodies triggers ITAM phosphorylation via Src family kinases, followed by Syk recruitment and assembly of the LAT–SLP-76 signalosome, activating PLCγ2, PI3K, and Btk-dependent amplification to drive calcium mobilization, integrin activation, degranulation, and procoagulant surface formation [104,276]. This enables formation of large platelet–leukocyte aggregates that immobilize pathogens and facilitate phagocytic clearance [277], and directly enables platelets to recognize and kill IgG-opsonized bacteria [278,279]. Pharmacologic Syk inhibition blocks this pathway [280]. Pathologically, FcγRIIa cross-linking drives systemic anaphylaxis [281] and underlies heparin-induced thrombocytopenia (HIT), where PF4–heparin complexes elicit pathogenic antibodies that activate platelets via FcγRIIa, causing thrombosis and thrombocytopenia [282]. The FcγRIIa H131R polymorphism influences IgG subclass binding and modifies thrombotic risk in HIT [283,284], and analogous FcγRIIa-dependent mechanisms are implicated in vaccine-induced immune thrombotic thrombocytopenia, though initiating antigens and risk factors remain incompletely defined [285].

Platelet CD40L provides direct co-stimulatory signals to B cells, enhancing their activation and antibody production [63,286], while PF4 promotes early B-lineage differentiation via STAT5 phosphorylation [287]. Platelets additionally store pathogen-specific IgG in α-granules and release it upon re-encountering the relevant pathogen—transfusion of influenza-experienced platelets into naïve mice conferred antibody-mediated immunity comparable to that of seropositive animals, demonstrating a form of platelet-mediated serological memory [288]. Notably, PF4 serves as an amplifier of these humoral interactions: released from α-granules, PF4 binds negatively charged polyanions on bacterial surfaces, opsonizing them, altering their conformation, and exposing neoepitopes that trigger anti-PF4/polyanion antibody formation [279]. The resulting immune complexes further activate platelets via FcγRIIa, enhancing bacterial clearance in a self-amplifying loop [279].

Therefore, platelets contribute to adaptive immunity across a remarkable functional breadth—structurally supporting lymphoid organ architecture, directing lymphocyte trafficking, presenting antigens, co-stimulating T and B cells, shaping T cell polarization, and mediating humoral immune surveillance through Fc receptor signaling and CD40L/PF4-dependent pathways. Together, these mechanisms establish platelets as active and indispensable participants in adaptive immune responses rather than passive bystanders, and set the stage for understanding how dysregulation of these same pathways contributes to inflammatory disease and tumorigenesis examined in the following section.

The receptor–ligand interactions described throughout Section 3, Section 4, Section 5.1 and Section 5.2, collectively define the mechanistic foundation of platelet immunologic function. Table 2 summarizes these key pairings alongside their immune partner cells and functional outcomes to provide an integrated reference before examining how dysregulation of these same pathways drives disease pathogenesis.

### 5.3. Platelet Contribution to Pathogenesis

#### 5.3.1. Chronic Inflammatory and Systemic Disease

The same immune capabilities that make platelets effective host defenders render them potent drivers of pathology when dysregulated. Platelets are uniquely positioned to amplify chronic inflammation by virtue of three characteristics: their abundance in circulation, their broad receptor repertoire capable of activating multiple immune pathways, and their capacity for rapid, sustained granule release [18]. When platelet activation is prolonged or insufficiently regulated, these properties drive immune-mediated inflammatory diseases (IMIDs) including rheumatoid arthritis, systemic lupus erythematosus, Sjögren’s syndrome, and systemic sclerosis [309,310,311]—conditions in which elevated circulating platelet–leukocyte complexes, platelet microparticles in lymphatics, and enhanced immunomodulatory receptor expression are consistently observed. Outside IMIDs, dysregulated platelet activation contributes to atherosclerosis, transplant rejection, and the chronic complications of malaria [168,312].

The mechanistic basis for platelet-driven chronicity centers on a self-reinforcing loop between granule release and sustained immune activation [17]. Prolonged TGF-β release from α-granules drives pro-fibrotic tissue remodeling—a mechanism particularly relevant in systemic sclerosis [313]— and vascular remodeling in sickle cell disease [314], while the pro-inflammatory milieu generated by sustained platelet activation may eventually overwhelm the regulatory capacity of TGF-β-induced immune checkpoints. In the acute phase of infection, vascular injury, or immune activation, platelets initiate protective immunothrombosis—a pathogen-restricting mechanism coordinated through IL-6 and IL-8 signaling from immune cells [107,315]. However, with continued platelet activation, this initially protective program becomes maladaptive: immunothrombosis transitions to systemic thrombo-inflammation, IL-6 and IL-8 drive progressive endothelial injury, and platelet consumption contributes to coagulopathy [316]. Targeting IL-6 and IL-8 signaling, or selectively dampening platelet immunoactivation at this inflection point, represents a rational but as yet incompletely realized therapeutic strategy [317].

#### 5.3.2. Immune Evasion and Tumorigenesis

Platelet–immune duality extends into the cancer context. In early or pre-malignant settings, platelets exert protective anti-tumorigenic effects through thrombospondin-1, an endogenous angiogenesis inhibitor stored in α-granules [318], and through P2Y_12_-dependent CD40L release that limits liver tumor growth in the context of NAFLD-associated hepatocellular carcinoma [319]. However, as tumors evolve, they exploit platelet–immune functions to promote immune evasion and metastasis, and these pro-tumorigenic roles are considerably better characterized.

Tumors actively reprogram circulating platelets into tumor-educated platelets (TEPs) through bidirectional cargo exchange, extracellular vesicle transfer, and receptor remodeling [320]. Upon activation, TEPs aggregate around circulating tumor cells to form microtumor thrombi that shield malignant cells from shear stress and NK cell cytolysis, while platelet-derived TGF-β suppresses NK cell reactivity by downregulating NKG2D and inhibiting the CD226/CD96–CD112/CD155 co-stimulatory axis [321]. Likewise, platelets impair adaptive anti-tumor responses by upregulating PD-L1 on cancer cells in an EGFR-dependent manner [322], and TEPs directly acquire PD-L1 via a fibronectin-1/GPIbα/integrin α5β1-dependent transfer mechanism—suppressing T cell immunity even in PD-L1-negative tumors, which may explain checkpoint inhibitor efficacy in patients whose tumors lack canonical PD-L1 expression [323,324]. In non-small cell lung cancer (NSCLC), tumor-associated platelets release TLT-1, which binds CD3ε and activates NF-κB to directly suppress CD8^+^ T cell function, with TLT-1 levels correlating inversely with patient survival [325]. Within the tumor microvasculature, tumor-released nucleic acids induce procoagulant platelet activation, generating activated platelets that deliver immune checkpoint molecules to suppress anti-tumorigenic lymphocytes while activating pro-tumorigenic myeloid leukocytes—disrupting these platelet–leukocyte interactions reduces tumor progression with efficacy comparable to systemic immunotherapy in orthotopic triple-negative breast cancer models [326].

Clinically, pretreatment thrombocytosis and elevated platelet counts consistently predict inferior survival across multiple malignancies [327,328], underscoring the translational relevance of these mechanisms. Platelet-derived indices refine this prognostic signal further: an elevated platelet distribution width-to-platelet ratio and red cell distribution width-to-platelet ratio are independent predictors of poor disease-free survival in breast cancer [329,330], while a higher preoperative platelet-to-albumin ratio independently may predict poor overall survival in NSCLC [331]. These findings converge with clinical evidence that concomitant aspirin use has been associated with extended survival in patients receiving immune checkpoint inhibitors [332], that adjuvant low-dose aspirin significantly reduces recurrence in resected colorectal cancer harboring PI3K pathway alterations—a finding now incorporated into NCCN guidelines recommending aspirin 100–162 mg daily for 3 years in stage II–III colorectal cancer with somatic PI3K alterations [333]—and that long-term aspirin use enhances immunotherapy efficacy in advanced NSCLC [334].

Taken together, platelets appear to support cellular immunity and suppress tumor growth in pre-malignant or early-cancer stages, while undergoing a functional pivot toward immunosuppressive and pro-tumorigenic roles in established advanced disease. This stage-dependent duality underscores the need to better characterize the molecular triggers of this transition and to develop targeted interventions capable of exploiting the anti-tumorigenic functions of platelets while neutralizing their pro-tumorigenic ones.

## 6. Conclusions and Future Directions

Platelets emerge from this review as dynamic immune sentinels whose canonical hemostatic machinery is repurposed to sense danger signals, shape inflammation, and coordinate host defense. Their rich repertoire of surface and intracellular receptors—including TLRs, integrins, selectins, GPVI, FcγRIIa, and CD40L—enables rapid detection of PAMPs, DAMPs, immune complexes, and vascular injury cues. Preformed mediators stored in α-, δ-, and λ-granules, together with plasma membrane-derived extracellular vesicles, allow for the temporally controlled release of chemokines, cytokines, growth factors, adhesion proteins, and microbicidal peptides. Through these mechanisms, platelets recruit and activate neutrophils, monocytes, macrophages, DCs, NK cells, and lymphocytes, bridging innate and adaptive immunity through antigen presentation, co-stimulatory signaling, and Fc receptor-mediated humoral surveillance. Conversely, unchecked platelet activation drives pathogenesis in sepsis, thrombo-inflammatory disorders, autoimmune diseases, and cancer—underscoring their remarkable functional breadth covered in this review.

The central challenge is that platelet–immune functions are bidirectional, context-dependent, pathogen-specific, and stage-dependent, making selective therapeutic targeting difficult. Current antiplatelet agents are blunt instruments that cannot discriminate between harmful and beneficial platelet–immune interactions. Several specific gaps warrant priority attention. First, in sepsis and critical illness, the precise temporal and molecular checkpoint at which protective immunothrombosis converts to pathologic thrombo-inflammation and DIC remains undefined—identifying this inflection point is a prerequisite to timed, context-specific intervention. Second, receptor-specific antagonists targeting GPVI for thrombo-inflammatory lung injury, FcγRIIa for HIT and immune complex-driven pathology, or discrete TLR isoforms for pathogen-specific modulation are needed to uncouple immune from hemostatic platelet function. Third, TEP RNA signatures require prospective validation as liquid biopsy biomarkers for cancer detection and immunotherapy response monitoring, and platelet-derived extracellular vesicles warrant systematic evaluation as engineered drug delivery platforms for immunomodulatory cargo in tumor microenvironments. Finally, integrating soluble platelet activation biomarkers—CD40L, PF4, P-selectin, and microparticle profiles—into clinical trial design will enable patient stratification and proof-of-concept testing of immune-focused antiplatelet strategies that preserve primary hemostasis. Until such precision tools are developed, antiplatelet therapy for immunomodulation will remain empiric rather than mechanism-guided.

## Figures and Tables

**Table 1:** Select platelet-derived inflammatory mediators and immune modulators.

Organelle	Mediator	Characteristic	Immune/Inflammatory Role
α-granules	CD40L	TNF superfamily	APC [157], B-cell responses [158], endothelial cell [159] activation
	CD63	Tetraspanin	Leukocyte recruitment [135]
	Cyclophilin A	Immunophilin	Ca^2+^, ROS production [160,161]
	MMP-2, MMP-9	Protease	Extracellular matrix breakdown, platelet-leukocyte aggregate formation [162]
	MIP-1α (CCL-3)	Cytokine	Neutrophil/eosinophil activation, B-cell Ig production [163,164]
	PDGF	Growth factor	Cell growth, monocyte/macrophage differentiation [165,166]
	PF4 (CXCL-4)	Chemokine	Monocyte, neutrophil [167], T-cell recruitment [168], Th differentiation [169,170]
	Ppbp β-thromboglobulin NAP-2 (CXCL-7)	Chemokine	Neutrophil activation and recruitment, macrophage phagocytic activity [171,172,173]
	CCL-5 (RANTES)	Chemokines	Recruitment of neutrophil [174,175], monocytes [176] and T cells [177]
	P-selectin	Selectin	Leukocyte adhesion [178,179], complement activation [180]
	Thrombospondins	Matricellular protein	Apoptosis [181], endothelial cell inflammation [182], macrophage-platelet aggregates [183]
	TGF-β	Cytokine	Cell proliferation, T-cell differentiation, B-cell/macrophage phenotype regulation [184,185,186,187,188,189]
	VEGF	Growth factor	Angiogenesis, adhesion molecule expression [190,191,192]
	vWF	Multimeric adhesive glycoprotein	Platelet adhesion, PMN extravasation [193,194]
Dense (δ−) granules	ADP	Purine nucleotide	Platelet, leukocyte and endothelial cell activation [195,196]
	Histamine	Biogenic amine	Increased vessel reactivity, degranulation, proinflammatory response [197,198,199,200]
	Serotonin	Biogenic amine	Monocyte, DC and T-cell function [201,202,203]
	Glutamate	Excitatory amino acid	T-cell trafficking [204,205]
	Polyphosphates	Inorganic polymer	Inflammatory response amplification [206]
Expressed/Produced	IL-1β	Cytokine	APR, leukocyte/endothelial activation [207,208]
	GPIbα	Adhesion molecule	Binds Mac-1 on leukocytes [209]
	Nitric oxide	ROS	Anti-inflammatory and anti-thrombotic [210]

Table abbreviations: APC, antigen presenting cell; NAP-2, neutrophil activating peptide 2; Ppbp, pro-platelet basic protein; VEGF, vascular endothelial growth factor; vWF, von Willebrand factor; ADP, adenosine 5′-diphosphate; TGF-β, transforming growth factor beta; IL-1β, interleukin-1 beta; GPIbα, glycoprotein Ibα; PDGF, platelet-derived growth factor; MMP, metalloproteinase; MIP-1α, macrophage-inflammatory protein; PF4, platelet factor 4; PMN, polymorphonuclear neutrophil; DC, dendritic cell; APR, acute phase response; ROS, reactive oxygen species.

**Table 2:** Key platelet receptor-ligand interactions and their immunologic roles.

Platelet Receptor	Ligand(s)	Immune Partner	Functional Outcome	Ref
GP1b-IX-V	vWF, Mac-1 (CD11b)	Neutrophils, DCs	Leukocyte tethering, pathogen shuttling to CD8α^+^ DCs	[264,289]
TLR2/1, TLR2/6	Bacterial lipopeptides	—	PI3K-driven aggregation, bacterial detection	[76,77]
TLR4	LPS (Gram-negative bacteria)	Neutrophils	NETosis, platelet–neutrophil aggregate formation	[78]
TLR7	ssRNA (influenza virus)	Neutrophils	C3 release, NET-driven acute lung injury	[86]
TLR9	Unmethylated CpG DNA	Neutrophils	Endosomal signaling, CXCL4-driven NETosis	[290]
CLEC-2	Podoplanin, dengue virus	DCs, lymphatics	Platelet aggregation, lymph node architecture maintenance	[66,291]
GPVI	Collagen, fibrin, laminin	Neutrophils, endothelium	ITAM activation, bacterial clearance, platelet-leukocyte aggregates, vessel repair	[106,292,293]
FcγRIIa (CD32a)	IgG immune complexes	Monocytes, neutrophils	Platelet–leukocyte aggregates, pathogen clearance, HIT, anaphylaxis	[281,294,295,296]
FcεRI	IgE	Mast cells (indirect)	Serotonin/RANTES release, allergic response amplification	[297,298,299]
PECAM-1 (CD31)	PECAM-1 (homophilic)	Endothelium, leukocytes	Leukocyte transmigration, ITIM-mediated inhibition of platelet activation	[300,301]
TLT-1	TREM-1 ligand	Neutrophils, monocytes	Dampens leukocyte activation in sepsis; promotes IL-10-producing B cells	[121,123]
P-selectin (CD62P)	PSGL-1	Neutrophils, monocytes, Tregs	Leukocyte rolling/recruitment, NETosis, Treg transcriptome modulation	[250,302,303]
αIIbβ3 (GPIIb/IIIa)	Fibrinogen, vWF, Mac-1	Neutrophils, DCs, B cells	PNC formation, NETosis, CD40L delivery, antigen presentation	[128,262,304]
PAR1	Thrombin, ssRNA virus	—	IFN-β induction, innate antiviral defense	[145]
PAR4	Thrombin	Leukocytes, Tregs	Pro-inflammatory granule release; PAR4 inhibition augments Treg activation	[146,305]
CD40L (CD154)	CD40	B cells, DCs, T cells	B cell co-stimulation, antibody class switching, DC maturation, T cell priming	[129]
PF4 (CXCL4)	CXCR3, polyanions	T cells, neutrophils, B cells	T cell recruitment, bacterial opsonization, anti-PF4 antibody formation	[306,307,308]

Table abbreviations: vWF, von Willebrand factor; DC, dendritic cell; TLR, toll-like receptor; LPS, lipopolysaccharide; NET, neutrophil extracellular trap; ssRNA, single-stranded RNA; CLEC-2, C-type lectin-like receptor 2; GPVI, glycoprotein VI; ITAM, immunoreceptor tyrosine-based activation motif; FcγRIIa, Fc gamma receptor IIa; HIT, heparin-induced thrombocytopenia; FcεRI, Fc epsilon receptor I; PECAM-1, platelet endothelial cell adhesion molecule-1; ITIM, immunoreceptor tyrosine-based inhibitory motif; TLT-1, TREM-like transcript-1; TREM-1, triggering receptor expressed on myeloid cells-1; PSGL-1, P-selectin glycoprotein ligand-1; PNC, platelet–neutrophil complex; NETosis, neutrophil extracellular trap release; PAR, protease-activated receptor; IFN-β, interferon-beta; CD40L, CD40 ligand; PF4, platelet factor 4.

## Data Availability

All relevant data is included in the manuscript.
